# Lifetime Fitness Variation Across the Geographical Range in a Colour Polymorphic Species

**DOI:** 10.1002/ece3.71051

**Published:** 2025-04-27

**Authors:** Gian Luigi Bucciolini, Chiara Morosinotto, Jon Brommer, Al Vrezec, Peter Ericsson, Lars‐Ove Nilsson, Karel Poprach, Ingar Jostein Øien, Patrik Karell

**Affiliations:** ^1^ Department of Biology University of Turku Turku Finland; ^2^ Department of Bioeconomy Novia University of Applied Sciences Tammisaari Finland; ^3^ Evolutionary Ecology Unit, Department of Biology Lund University Lund Sweden; ^4^ Department of Biology University of Padova Padua Italy; ^5^ National Biodiversity Future Center (NBFC) Palermo Italy; ^6^ Department of Organisms and Ecosystems Research National Institute of Biology Ljubljana Slovenia; ^7^ Slovenian Museum of Natural History Ljubljana Slovenia; ^8^ Biotechnical Faculty University of Ljubljana Ljubljana Slovenia; ^9^ Fagersanna Sweden; ^10^ Karlsborg Sweden; ^11^ TYTO, z. s. Věrovany Czech Republic; ^12^ Faculty of Science Palacky University Olomouc Czech Republic; ^13^ BirdLife Norway Trondheim Norway; ^14^ Department of Ecology and Genetics University of Uppsala Uppsala Sweden

**Keywords:** colour polymorphism, fitness, geographical range, life‐history traits, reproductive investment

## Abstract

The maintenance of variation (i.e., different phenotypes) for heritable traits that are under selection, despite expectations of selection eroding variation and favouring only the fittest phenotype, represents an evolutionary paradox. Here, we studied variation in life‐history traits in five populations of colour polymorphic tawny owls ( *Strix aluco* ) across Europe that have been individually studied for 13 years. Tawny owls show heritable plumage colour variation ranging from less pigmented (grey) to more heavily pigmented (brown‐red). The breeding life span (BLS), lifetime egg production (LEP), lifetime reproductive success (LRS) and the number of years skipped between breeding attempts (NYS) varied between the study populations, with LEP and LRS varying across colour morphs in a population‐specific fashion. Thus, grey tawny owl females have higher lifetime fledgling and egg production than brown‐red females in some populations, but vice versa in others. Hence, our findings demonstrate disruptive selection of tawny owl plumage colourations across their European range, which may be one factor maintaining variation in heritable tawny owl colourations.

## Graphical abstract

Wide geographical‐scale investigation of life‐history among different tawny owl plumage colour phenotypes across Europe. Our findings highlight the complexity of reproductive strategies between different populations and within single populations over large spatial scales, suggesting that factors influencing eggs and fledglings production vary across different regions.

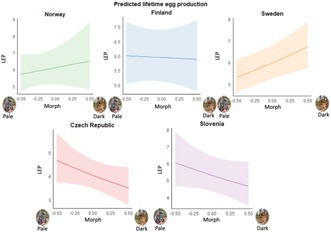

## 1 Introduction

The living world is marked by striking variations in various traits exhibited by different organisms. Such traits may represent, among others, morphological, behavioural and physiological aspects that often covary with demographic properties (e.g., reproduction rate, survival within reproductive events, early or late fecundity, adult size and mortality) of an organism that determine its fitness (Rose and Mueller 1993; Heino et al. 1997). If a trait is heritable and causes fitness differences, selection is expected to erode its variation (Ellegren and Galtier 2016; Gillespie 1991; Fisher 1999). A central question is therefore how variation (polymorphy) is maintained in nature in the face of natural selection acting on it. In many organisms, different morphs are characterised by a complex of genes that express a certain phenotype associated with a specific life‐history strategy, for example, in insects (Ahnesjö and Forsman 2003; Soares et al. 2001), fish (Hutchings and Myers 1994), reptiles (Galeotti et al. 2013) and birds (Brommer et al. 2005; San‐Jose et al. 2019; Tuttle 2003), and this confers higher fitness to the individuals in certain conditions. From that perspective, disruptive selection, where different morphs are selectively favoured over environments varying across time and/or space, could be one process maintaining variation in nature (Galeotti et al. 2003; Michie et al. 2010; Robinson et al. 2024).

The colour polymorphic tawny owl ( *Strix aluco* ) is an excellent candidate to investigate whether selection acts differently on morphs across the species’ range. In this nocturnal raptor, the plumage colouration is determined by the degree of pheomelanin irreversibly deposited in the plumage, and this colouration is highly heritable and independent of age and sex (Brommer et al. 2005). Indeed, tawny owls display a range of plumage colours, ranging from pale grey to brown‐red, that are generally classified into two morphs: a grey and a brown morph (Galeotti and Cesaris 1996; König et al. 1999). Colour morphs in tawny owls have been associated with different physiological and behavioural traits, and are thus likely to correlate with diverse fitness (Brommer et al. 2005; Emaresi et al. 2014; Karell et al. 2011a, 2013, 2021; Koskenpato et al. 2016; Morosinotto et al. 2020; Piault et al. 2009). The tawny owl has a widespread distribution across Europe and therefore lives under different environmental and climatic conditions across its range (Ratajc et al. 2023). In southern Finland, grey individuals survive longer and produce more offspring than brown‐red ones during their lifetime (Brommer et al. 2005). Additonally, in this population, there is climate‐driven selection against brown‐red owls when winters are colder and more snow—rich, whereas when winters are milder, both morphs survive equally well (Karell et al. 2011a). This finding implies that across its range, selection would favour different tawny owl morphs. Indeed, it has been shown that their spatial distribution is affected by general climatic conditions (Koskenpato et al. 2023).

Here, we conduct a distribution‐wide geographical‐scale investigation of life‐history traits among different tawny owl plumage colour phenotypes across five local populations in Europe. We compared some of the most important life‐history traits for the tawny owl's fitness in order to explore if there were differences among the morphs across the species' range, considering different populations settled under different environmental conditions. In each population, individually marked females were colour scored, and their reproductive output was monitored for 13 years. This long‐term data allow us to compute breeding lifespan (hereafter BLS), lifetime egg production (hereafter LEP), lifetime reproductive success (hereafter LRS) and the number of years skipped between breeding attempts (hereafter NYS) as estimates of individual fitness in these tawny owl populations. We expect the direction of selection on plumage colouration to differ across populations, with darker tawny owls having reduced fitness components relative to light ones in some populations, but vice versa in other populations. In previous studies, it has been shown that in Finland, grey individuals have higher fitness than brown ones (Brommer et al. 2005) but vice versa in Switzerland (Emaresi et al. 2014) and these differences might be driven by the environmental conditions in which these populations are settled (Karell et al. 2011a; Gangoso and Figuerola 2019; Tate and Amar 2017). Hence, we also expect that environmental drivers might affect the morph frequency, underlying one of the potential mechanisms for the development and maintenance of different phenotypes. This work improves our understanding of the persistence of phenotypic variation in heritable traits under selection, especially in colour polymorphic species such as tawny owls.

Additionally, in this study, we present a new method that allows us to compare and align different colour‐scoring techniques, providing a valid approach for merging different datasets. This method (see ‘Assessment of plumage colouration’) is particularly useful for studying species within a large geographical range and enhancing temporal resolution when data are collected differently, offering a strong framework for such cases in colour polymorphic species.

## 2 Material and Methods

### 2.1 Study Populations and Methods

**FIGURE 1:**
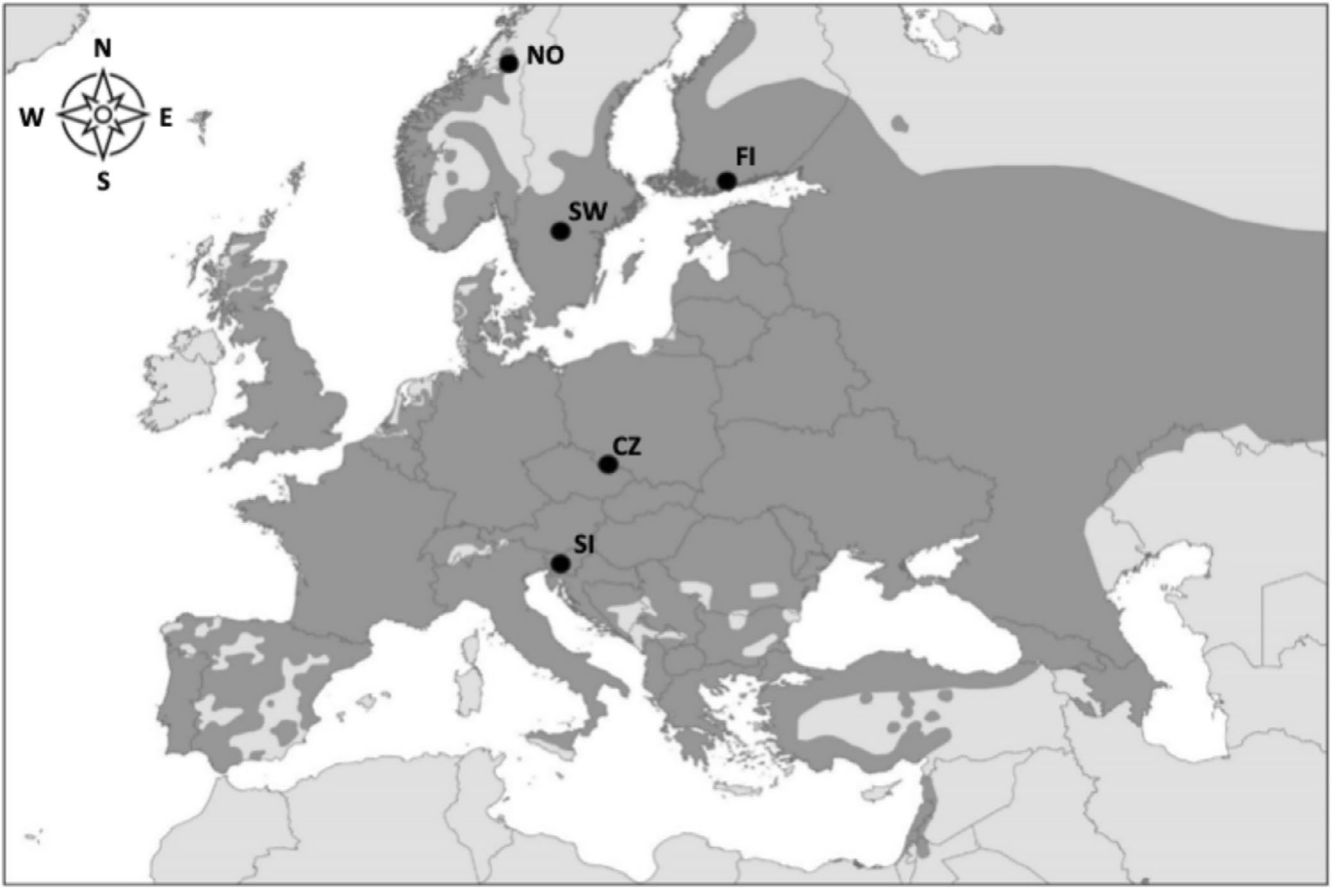
Distribution range of tawny owls in Europe (grey shaded) with points of study sites (figure prepared by Andrej Kapla).

The study was conducted in five nest box breeding populations of tawny owls (Figure 1). Three populations were located on the northern limit of the species range: Finland (central point Siuntio: 60°15′ N, 24°15′ E; hereafter FI), Sweden (central point Undenäs: 58°39′ N 14°25′ E; hereafter SW) and Norway (central point Levanger: 63°43′ N, 11°21′ E; hereafter NO), whereas the other two populations considered were in central/south Europe: Czech Republic (central point Olomouc: 49°69′ N, 17°15′ E; hereafter CZ) and Slovenia (central point Mt. Krim: 45°58′ N, 14°25′ E; hereafter SI).

These study areas are located at different latitudes and altitudes and are thus characterised by different climatic and environmental conditions. In the north of Europe (FI, SW and NO), at the limit of its range, the tawny owl lives and breeds in a variety of habitats, like agricultural areas and spruce‐dominated or mixed forests, whereas in the southern populations (SI and CZ) the main habitat is the broadleaf and mixed forest, but also agricultural and urban areas are frequently occupied. In SI, the study area is all covered with continuous mixed mountain forest where the dominant species are beech and fir (genera *Abies*, *Acer*, *Fagus* and *Picea*; Vrezec and Tome 2004). In the Czech Republic, the study area is mainly composed of forest complexes accompanied by a mosaic of agricultural landscapes (which is more represented in lower altitudes).

All the data considered in this study were based on populations of tawny owls breeding in nest boxes, with annual monitoring of breeding events. Nests were found by checking the boxes in spring in all the study populations when clutch size (number of eggs) was counted; laying date, however, varied across populations according to latitude. In cases where there were nestlings present at the first check, we assumed the clutch size was the sum of unhatched eggs and nestlings. The number of nestlings ringed (25–30 days posthatch) was considered to be the reproductive output. In all populations, female tawny owls were trapped during breeding and individually identified using a metal ring. We included data on females' reproduction during a 13‐year time interval (2008–2020). Males were excluded due to incomplete data across populations. Although considering only females might have introduced a slight bias in the results because sex could have an interaction with morph, in our study system it has been demonstrated that clutch size is determined by females, while males only influence the timing of egg laying (Brommer et al. 2015). The monitoring of individuals in each population started prior to 2008 and continued until (or after) 2022. However, for the analyses, we restricted the data to this 13‐year period in order to include only females that presumably started and ended their breeding career during the Years 2008–2020. That is, only the females that were caught for the first time during this time interval (2008 or later) and had not bred in the last 2 years of the datasets (2021 and 2022) were included to get information on lifetime reproduction.

Tawny owls, like other raptors, do not necessarily breed each year (Roulin et al. 2003). We calculated breeding lifespan (BLS) as the number of years between the first and last recorded breeding attempt of a female, independently of whether the individual bred continuously or skipped breeding between some attempts. BLS is a good estimation for survival in tawny owls because they are year‐round territorial, with high site tenacity and mate fidelity (Francis and Saurola 2004; Sunde 2011; Passarotto et al. 2023), while breeding dispersal occurs very seldom (Passarotto et al. 2023). BLS has been calculated, and not the total lifespan, because for most tawny owls, the exact age at first breeding could not be estimated. Additionally, for most of the individuals in our data, it was not possible to calculate the complete lifespan because most individuals were not ringed as chicks but only as adults (at least 1 year old). We also calculated the lifetime egg production (LEP) as the sum of all eggs a female produced, and the lifetime reproductive success (LRS) as the sum of nestlings in the nest just before fledging that each female produced in their lifetime. Since these owls can skip breeding if the environmental conditions are not suitable, we also investigated the number of years skipped between reproductive events (NYS). For those females that bred only once, we assumed NYS is 0 because there is no interval between breeding attempts. A priori decisions were made to simplify the datasets and to minimise assessment errors of the variables of interest. Individuals that had missing information on clutch and brood size for some or all years were removed and not considered for LEP and LRS. Hence, out of the total individuals (*n* = 727), all of them were considered for the BLS and NYS analyses, whereas 692 were considered for the LEP analysis and 638 for the LRS analysis. The response variables for the CZ population might be slightly underestimated due to variations in capture effort, which was not as consistent as in the other study populations (FI, SWE, NO and SL).

### 2.2 Assessment of Plumage Colouration

The study populations were colour scored using different systems where several morphs were identified. In the FI population, the breeding tawny owls have been colour scored from 1978 onwards using a semicontinuous scale (Karell et al. 2011a), that is based on scoring the level of pheomelanin (degree of reddish‐brown colouration) on four different parts of the plumage: the facial disc (proportion of reddish‐brown feathers in the facial disc, 0%–100%/1–3 points), back (1–4 points), breast (1–2 points) and on general appearance (1–5 points; Brommer et al. 2005). The obtained colour score can vary from 4 (a pale grey‐coloured individual with very little reddish‐brown pigments) to 14 points (an intensively red‐coloured individual). The colour score distribution is bimodal, and the two morphs can be classified into a grey and a brown morph at the cut point between 9 and 10 (Brommer et al. 2005; Karell et al. 2011a). The other populations (SW, NO, CZ and SI) were originally colour scored using a different system where several morphs were identified (Table 2).

In Sweden, it was used the same colour‐scoring method that has been used in Finland but the individuals were grouped into more categories (Table 2). In the CZ population, three basic morphs were recognised: grey, brown and rusty, and a total of nine morphs were described and used for identification. For distinguishing individual morphs, we used colouration of the facial disc, back, wings and tail (Table 2). In Norway, the colour of each individual was assessed on the facial disc and the back. We distinguished five different colour variations from pale grey‐coloured individuals via brown, warm‐brown, red‐brown, to red. The colour of each individual was documented every year by photograph with the facial disc and back visible (Table 2). In the Slovenian population only three morph types were differentiated, two extremes, grey (or light) and red (or dark), with an intermediate brown morph. The scoring was conducted in the field during nest inspections, but additional photos of the facial disc have been taken for each female each year to enable further more detailed scoring (Table 2).

Spearman rank correlation was calculated for each population in order to show the repeatability of the original colour score method that has been used in the different countries (Altman 1990; Landis and Koch 1977). The Spearman correlation was computed between the colour scores assigned to individuals for each population in their first and second breeding attempts (for those individuals recorded at least twice). In SI, CZ and FI, the colour scoring was very consistent (*r* > 0.7; Table 1), whereas in NO and SW, the colour scoring was less consistent (*r* = 0.35; Table 1).

**TABLE 1:** Spearman's rank correlation test performed to evaluate the consistency of the original colour score method used in the study populations between the first and second breeding attempts of the individuals that have bred at least two times in the interval of time taken into account.

Population	Sample size	Spearman's rho	*p*
CZ	36	0.99	**< 0.0001**
FI	30	0.74	**< 0.0001**
NO	18	0.35	0.15
SI	12	1	**< 0.0001**
SW	222	0.35	**< 0.0001**

*Note:* Values in bold are significantly significant (*p* < 0.05).

**FIGURE 2:**
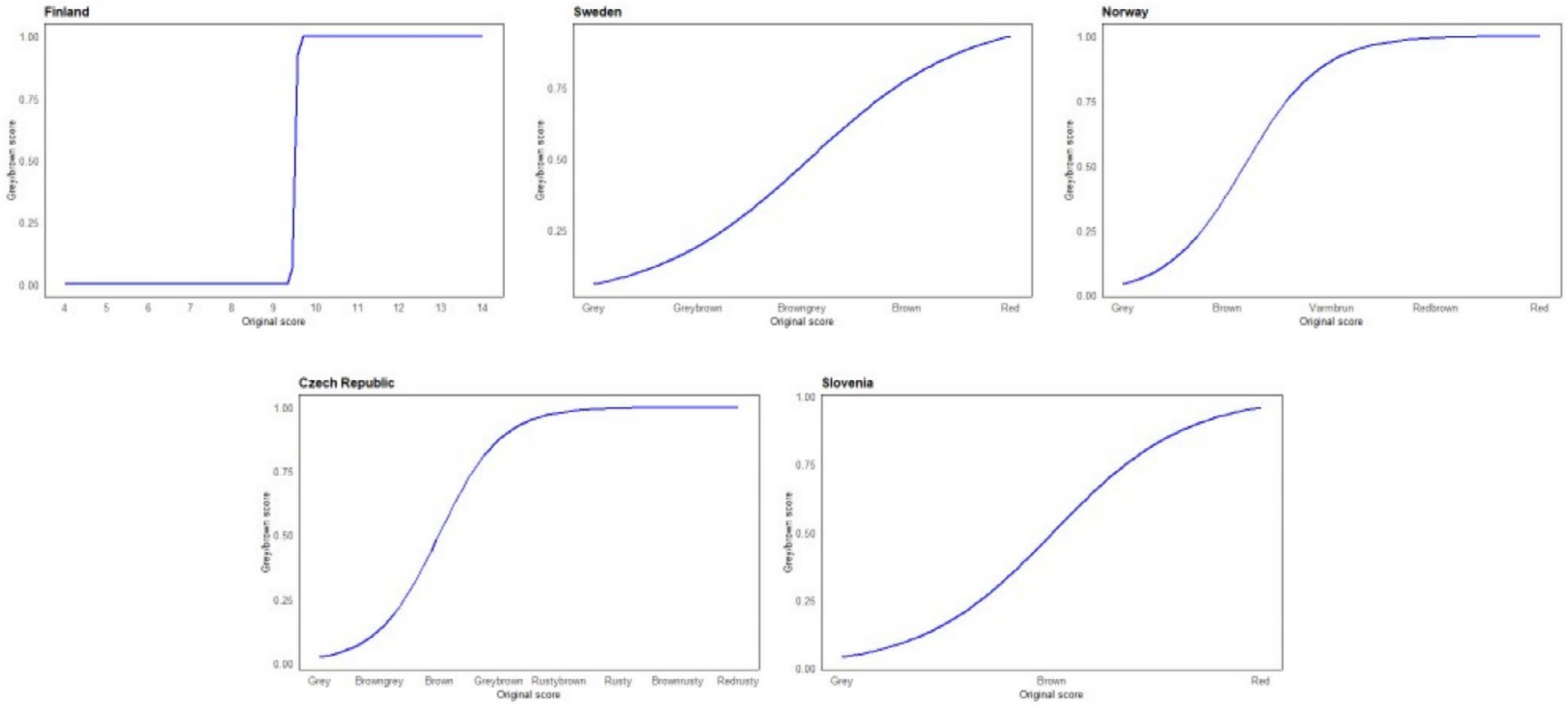
Plots of the logistic regression of bimodal (grey or brown) colour score based on pictures against the original (field‐based) colour score for each study population; the sample size for each population is as follows: FI: *n* = 117; SW: *n* = 51; NO: *n* = 66; CZ: *n* = 18; and SI: *n* = 39.

Pictures of the tawny owls from each population were used to compare the method of colour scoring originally used in the different populations with a bimodal classification of morphs as scored by an independent observer (GLB). We then regressed the original population‐specific score to this bimodal classification of colour. This cross‐population comparison shows that in each population, colour varies from the lightest individuals (pale; Figure 2) to the darkest ones (reddish; Figure 2) with different intermediate shades in between (Figure 2).

To include the different colour scores from all the populations in one analysis, the original colour scores were standardised. Distinct morphs were treated as varying colour shades, ranging from the lightest to the darkest within each population. To normalise these scores across populations with varying colour shades, a standardisation formula was applied: (*Score assigned based on colour shade—1*)/(*Maximum score—Minimum score*). This approach ensured that standardised values between 0 (palest individuals) and 1 (most reddish‐brown individuals) were obtained for each morph within and between populations (Table 2), allowing us to develop a unique method in which different colour score approaches are combined together.

**TABLE 2:** Summary of the colour score systems (original colour score) used in each population [Finland (FI), Sweden (SW), Norway (NO), Czech Republic (CZ) and Slovenia (SI)], arranged from the lightest to the darkest colour shade and the standardised values that we obtained to compare the study populations.

Population (*N*)	Original score (*N*)	Standardise values
FI (117)	4 (29)	0
	5 (12)	0.1
	6 (13)	0.2
	7 (8)	0.3
	8 (5)	0.4
	9 (6)	0.5
	10 (3)	0.6
	11 (5)	0.7
	12 (12)	0.8
	13 (18)	0.9
	14 (6)	1
SW (361)	Grey (60)	0
	Grey‐brown (43)	0.1428
	Brown‐grey (11)	0.2857
	Brown (195)	0.4285
	Brown‐red (1)	0.5714
	Red (11)	0.7142
	Reddish‐brown (23)	0.8571
	Rusty‐red (17)	1
NO (64)	Grey (15)	0
	Brown (37)	0.25
	Warm‐brown (3)	0.50
	Red‐brown (5)	0.75
	Red (4)	1
CZ (151)	Grey (3)	0
	Brown‐grey (26)	0.1428
	Brown (16)	0.2857
	Grey‐brown (18)	0.4285
	Rusty‐brown (37)	0.5714
	Rusty (22)	0.7142
	Brown‐rusty (25)	0.8571
	Red‐rusty (4)	1
SL (34)	Grey (9)	0
	Brown (12)	0.5
	Red (13)	1

*Note:* The number of individuals considered within each population and for each original score between 2008 and 2022 is presented in parentheses. Not all of these individuals were used for all the analyses (see *Study populations and methods*).

### 2.3 Statistical Analysis

All the analyses were run in R 4.3.1 (www.r‐project.org). GLMMs were conducted using ‘glmmTMB’ (version 1.1.5) package (Bolker et al. 2009). The response variables considered were BLS, LEP, LRS and NYS, which were count variables assumed to follow a Poisson distribution. Morph has been considered a continuous variable from the lightest (pale individuals) to the darkest colour shade (reddish individuals, see *Assessment of plumage colouration*), whereas population was considered a factorial fixed effect. In all the models, the interaction population*morph was included to test if there were differences among the study populations in the relationship (slope) of the response variables as a function of morph. The year the individual first bred was considered a random factor to control for the nonindependence of individuals that started breeding in the same year. Additionally, the Bonferroni correction has been applied to the models to account for multiple comparisons.

## 3 Results

**FIGURE 3:**
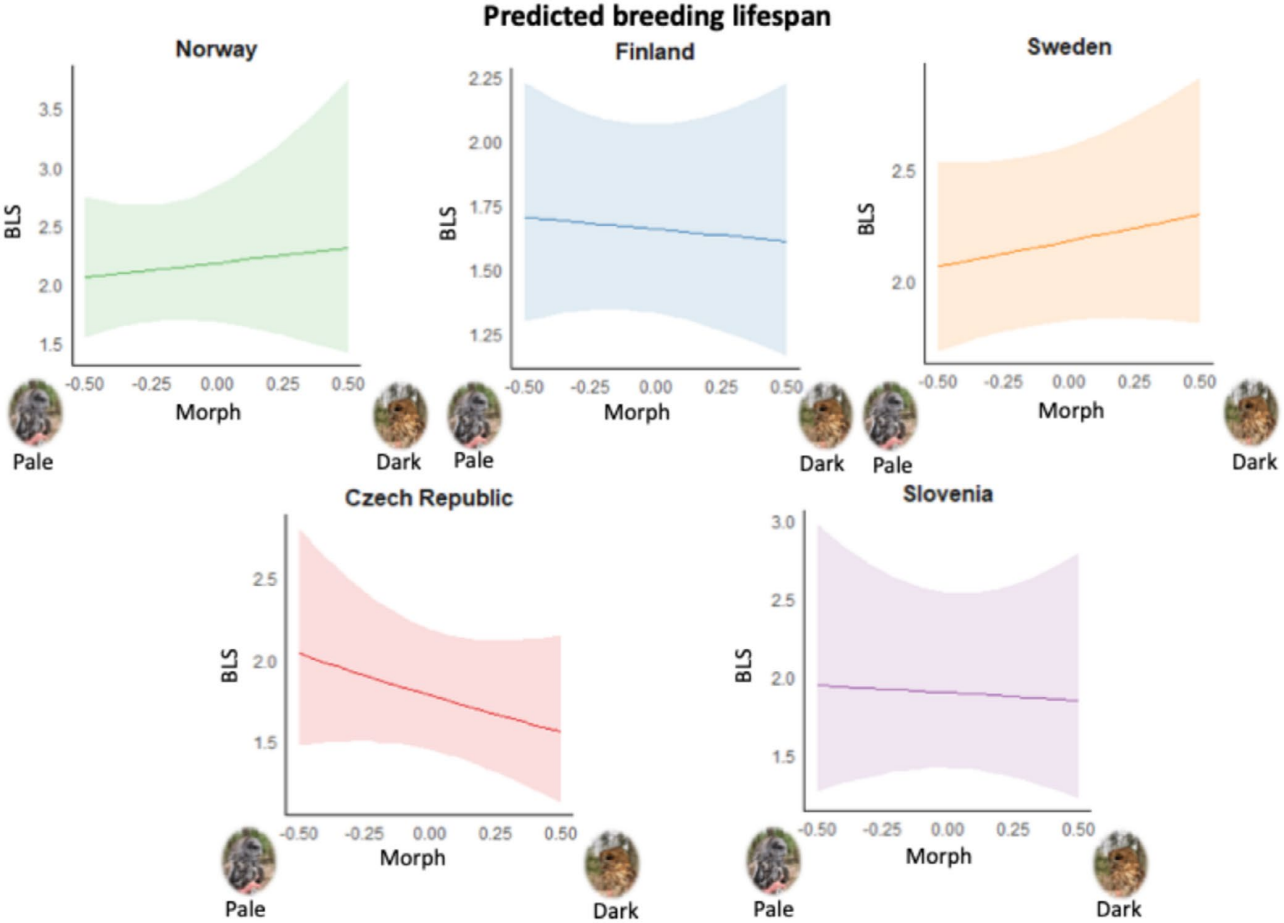
Estimates ± 95% CI of the BLS for the interaction of country and morph. Morph goes from the lightest (values on the left) to the darkest individuals (values on the right).

Most of the individuals had a BLS of 1 year (470 out of 727), with 13 years as the maximum BLS. Although tawny owls are known to live up to 20 years, such long lifespans were not recorded in our data. Of the 257 individuals that bred more than once, there were 119 tawny owls that did not skip any years between breeding attempts, while 138 individuals skipped at least one breeding season. While practically all individuals that skipped breeding during their life only had 1 or 2 years between breeding attempts, there were two individuals that returned to breeding in the territory after 8 years. We cannot rule out the possibility that these two individuals may have reproduced in other locations during this time, outside the monitored boxes. Regarding LEP, most of the individuals (459 of 692) laid between 1 and 5 eggs in their lifetime, 5 females produced no eggs and 2 individuals reached a maximum of 32 eggs. For the LRS (total sample size *n* = 638), 407 owls produced between 1 and 5 fledglings, 92 females did not produce any fledglings and 4 females produced between 20 and 25 fledglings in their lifetime. Clear variation in life‐history traits was observed throughout the species range. BLS differed between populations but did not vary with tawny owl plumage colouration (Table 3 and Figure 3).

**TABLE 3:** GLMM on the response variables (BLS and LEP) across Europe according to populations and morphs (from the lightest to the darkest colour shade).

ANOVA test				
Resp. variables	Ind. variables	*χ* ^2^	Df	*p*
BLS	**Country**	75.76	5	**< 0.0001**
	Morph	0.02	1	0.90
	Country*Morph	1.99	4	0.73
LEP	**Country**	777.55	5	**< 0.0001**
	Morph	1.39	1	0.23
	Country*Morph	12.26	4	**< 0.05**

*Z* test					
Resp. variables	Ind. variables	Estimate beta ± SE	Df	*Z*	*p*
BLS	**Country CZ**	0.57 ± 0.10	5	5.51	**< 0.0001**
	**Country FI**	0.50 ± 0.11	5	4.48	**< 0.0001**
	**Country NO**	0.78 ± 0.13	5	5.83	**< 0.0001**
	**Country SI**	0.64 ± 0.14	5	4.34	**< 0.0001**
	**Country SW**	0.78 ± 0.09	5	8.49	**< 0.0001**
	Morph	−0.26 ± 0.25	1	−1.06	0.28
	Country FI*Morph	0.21 ± 0.25	4	0.32	0.51
	Country NO*Morph	0.38 ± 0.39	4	0.39	0.33
	Country SI*Morph	0.21 ± 0.39	4	0.39	0.58
	Country SW*Morph	0.37 ± 0.28	4	0.28	0.18
LEP	**Country CZ**	1.39 ± 0.07	5	18.46	**< 0.0001**
	**Country FI**	1.78 ± 0.07	5	23.58	**< 0.0001**
	**Country NO**	1.80 ± 0.08	5	20.25	**< 0.0001**
	**Country SI**	1.66 ± 0.09	5	17.00	**< 0.0001**
	**Country SW**	1.79 ± 0.06	5	27.19	**< 0.0001**
	Morph	−0.28 ± 0.16	1	−1.69	0.08
	Country FI*Morph	−0.26 ± 0.20	4	1.28	0.19
	Country NO*Morph	0.41 ± 0.25	4	1.64	0.09
	Country SI*Morph	0.02 ± 0.25	4	0.10	0.92
	Country SW*Morph	0.52 ± 0.18	4	2.77	**< 0.01**

*Note:* Values in bold are statistically significant (*p* < 0.05), and values in bold and underlined are statistically significant also after having applied the Bonferroni correction (*p* < 0.0125). For each population the temporal range considered is 13 years (from 2008 to 2020); the sample size for each response variable for each population is as follows: BLS: CZ *n* = 151, FI *n* = 117, NO *n* = 158, SI *n* = 34 and SW *n* = 368; LRS: CZ *n* = 113, FI *n* = 103, NO *n* = 128, SI *n* = 32 and SW *n* = 350.

**FIGURE 4:**
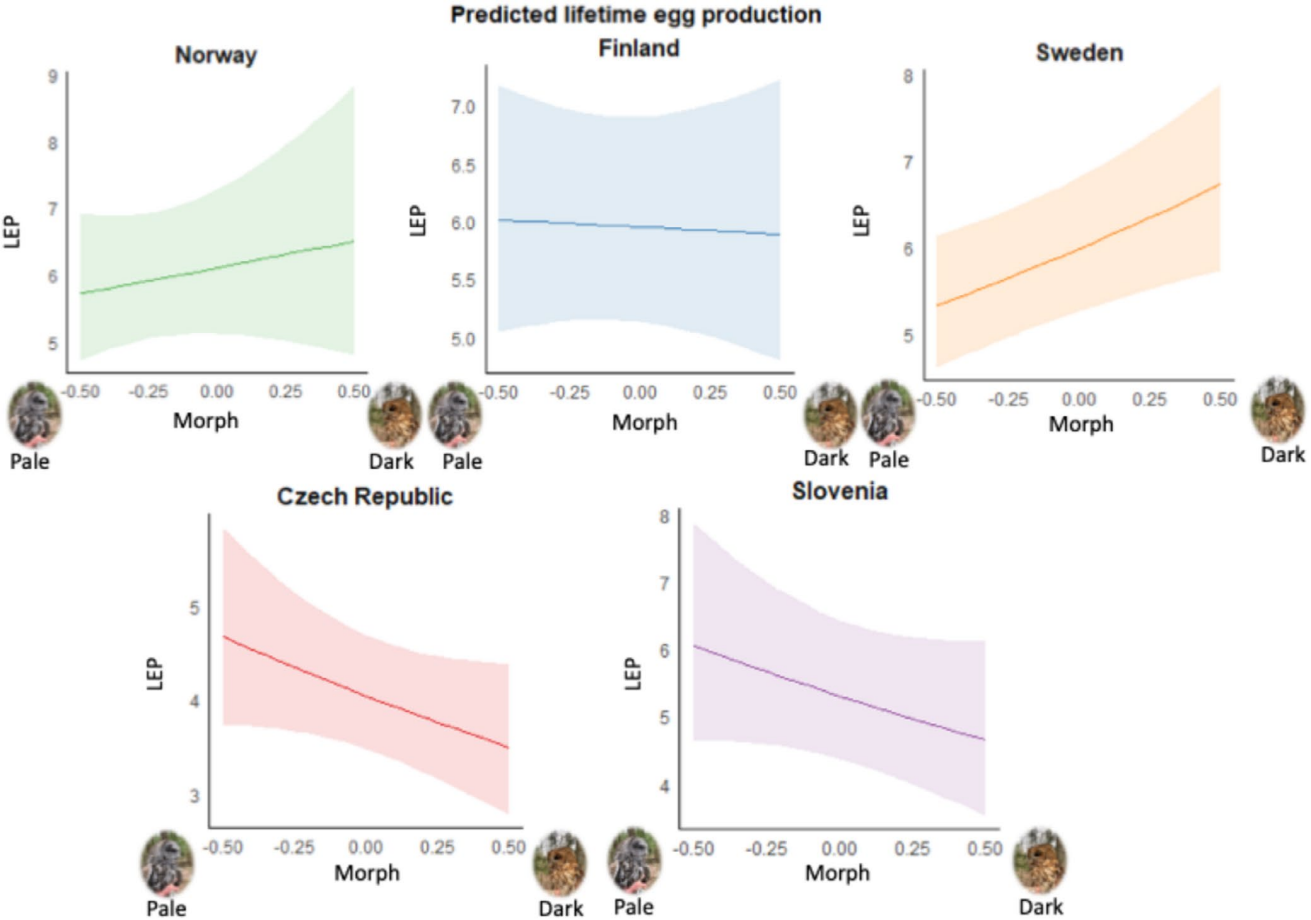
Estimates ± 95% CI of the LEP for the interaction of country and morph. Morph goes from the lightest (values on the left) to the darkest individuals (values on the right).

**FIGURE 5:**
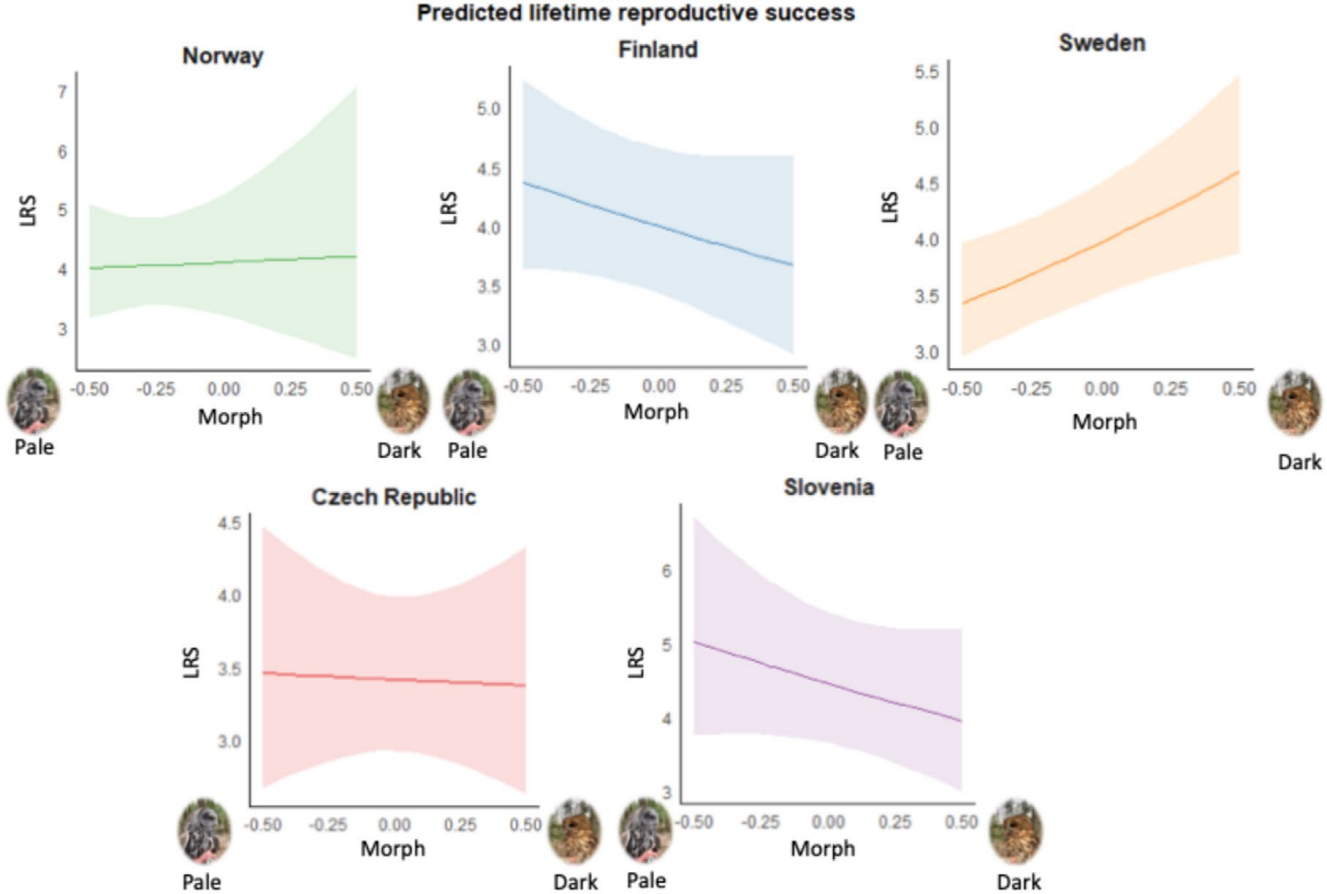
Estimates ± 95% CI of the LRS for the interaction of country and morph. Morph goes from the lightest (values on the left) to the darkest individuals (values on the right).

LEP and LRS are correlated (*r* = 0.77; *p* < 0.0001). LEP and LRS differed between populations (‘Country’ in Tables 3 and 4) but also depended in a population‐specific manner on morph (significant interaction ‘Country*Morph’ in Tables 3 and 4; see also Figures 4 and 5). Nevertheless, this interaction does not reach the significance threshold in both LEP and LRS when applying Bonferroni corrections, accounting for four tests that were conducted. Grey tawny owls had higher lifetime egg production compared to brown tawny owls in CZ and SI, while the opposite was observed in NO and SW (Figure 4). In FI, there are no big differences in the number of eggs laid during a lifetime between the morphs (Figure 4 and Table 3).

**TABLE 4:** GLMM on the response variables (NYS and LEP) across Europe according to populations and morphs (from the lightest to the darkest colour shade).

ANOVA test				
Resp. variables	Ind. variables	*χ* ^2^	Df	*p*
NYS	**Country**	27.84	5	**< 0.0001**
	**Morph**	3.92	1	**< 0.05**
	Country*Morph	5.50	4	0.23
LRS	**Country**	504.56	5	**< 0.0001**
	Morph	1.07	1	0.30
	Country*Morph	10.76	4	**< 0.05**

*Z* test					
Resp. variables	Ind. variables	Estimate beta ± SE	Df	*Z*	*p*
NYS	**Country CZ**	−1.35 ± 0.39	5	−3.42	**< 0.0001**
	**Country FI**	−2.18 ± 0.44	5	−4.90	**< 0.0001**
	**Country NO**	−1.29 ± 0.43	5	−2.93	**< 0.01**
	**Country SI**	−1.29 ± 0.45	5	−2.86	**< 0.01**
	**Country SW**	−1.17 ± 0.37	5	−3.08	**< 0.01**
	**Morph**	−1.62 ± 0.61	1	−2.62	**< 0.01**
	Country FI*Morph	0.84 ± 0.91	4	0.92	0.51
	Country NO*Morph	1.44 ± 0.91	4	1.57	0.33
	Country SI*Morph	1.91 ± 0.90	4	2.10	**< 0.05**
	Country SW*Morph	1.33 ± 0.68	4	1.96	**< 0.05**
LRS	**Country CZ**	1.22 ± 0.07	5	15.59	**< 0.0001**
	**Country FI**	1.38 ± 0.07	5	17.73	**< 0.0001**
	**Country NO**	1.41 ± 0.12	5	11.15	**< 0.0001**
	**Country SI**	1.49 ± 0.10	5	14.78	**< 0.0001**
	**Country SW**	1.37 ± 0.06	5	21.28	**< 0.0001**
	Morph	−0.02 ± 0.20	1	−0.11	0.90
	Country FI*Morph	−0.15 ± 0.24	4	−0.61	0.54
	Country NO*Morph	0.07 ± 0.38	4	0.18	0.85
	Country SI*Morph	−0.21 ± 0.29	4	−0.73	0.46
	Country SW*Morph	0.32 ± 0.22	4	1.41	0.15

*Note:* Values in bold are significantly significant (*p* < 0.05), and values in bold and underlined are statistically significant after having applied the Bonferroni correction (*p* < 0.0125). For each population, the temporal range considered is 13 years (from 2008 to 2020); the sample size for each response variable for each population is as follows: NYS: CZ *n* = 151, FI *n* = 117, NO *n* = 158, SI *n* = 34, SW *n* = 368; LEP: CZ *n* = 113, FI *n* = 103, NO *n* = 128, SI *n* = 32, SW *n* = 350.

**FIGURE 6:**
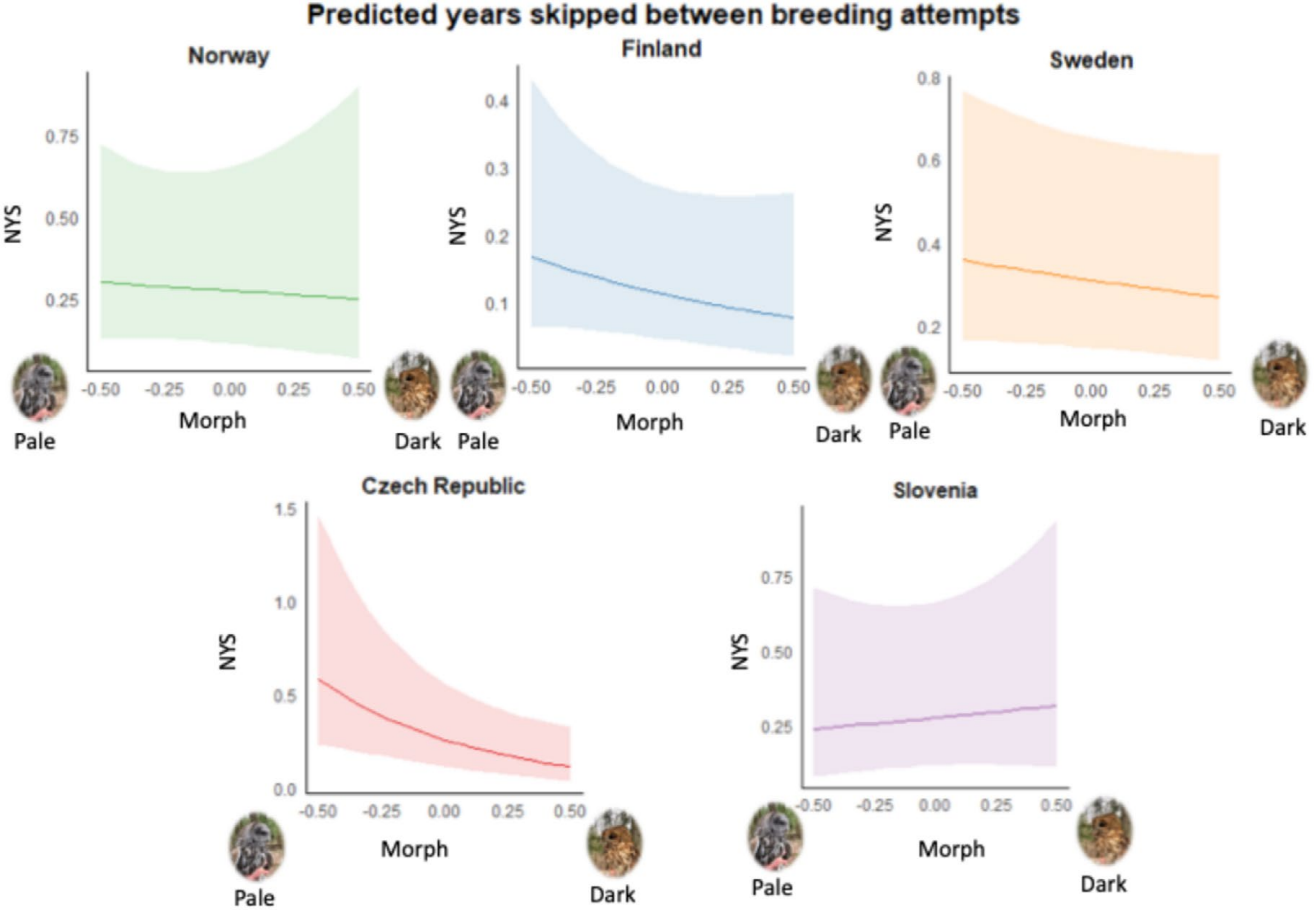
Estimates ± 95% CI of the NYS for the interaction of country and morph. Morph goes from the lightest (values on the left) to the darkest individuals (values on the right).

Populations (‘Country’) and morphs differed in NYS, but NYS did not depend on morph in a population‐specific manner (Figure 6 and Table 4). Overall, grey owls skip more years than the brown‐reddish ones between breeding attempts (Table 4), although it should be noted that morph does not reach the Bonferroni‐corrected significance threshold.

## 4 Discussion

Tawny owl plumage colouration is under selection, at least in some populations (Brommer et al. 2005; Emaresi et al. 2014), begging the question of how such polymorphism can be maintained. Previous studies suggest that distinct morphs are correlated with various traits that confer different advantages depending on the environmental context (Brommer et al. 2005; Tuttle 2003; Robinson et al. 2024; Emaresi et al. 2014; Karell et al. 2011a, 2013, 2021; Koskenpato et al. 2016; Morosinotto et al. 2020), with balancing selection acting on multiple traits to maintain morph frequency. Here, we show that there is no general pattern where one tawny owl colour morph is favoured across all five populations covering the central to northern European part of the range of these owls. However, despite the lack of a generalised morph‐specific pattern, there is variation in some of the main life‐history traits between and within the study populations. LEP and LRS varied across these study populations, and they depend on morph in a country‐specific manner. Pale (less pigmented) tawny owls produced more eggs (and fledglings) during their lifetime than brown‐reddish ones in some populations, whereas it was vice versa in other populations.

Our finding that different tawny owl plumage colour morphs have different fecundity across Europe is in line with previous works on colour polymorphism (Brommer et al. 2005; Karell et al. 2011a, 2013, 2021; Koskenpato et al. 2016; Morosinotto et al. 2020; Krüger 2002; Roulin 2004a, 2004b; Sgrò et al. 2013; Scali et al. 2015; Wit et al. 2015; Avilés et al. 2023). In general, these studies suggest that, due to differences in physiology, behaviour and life‐history traits related to melanin‐based colour polymorphism, distinct morphs are adapted to different environments (Roulin 2004a). These environments include not only the habitat itself but also biotic and abiotic factors that may influence behavioural and physiological traits (Robinson et al. 2024). For instance, the tawny owl grey morph seems to be better adapted to cold climates (Baltazar‐Soares et al. 2024). Clearly, environmental (or ecological) factors strongly shape this variation (Sgrò et al. 2013; Wit et al. 2015; Schmidt and Paaby 2008). For example, in common buzzards ( *Buteo buteo* ) environmental traits like habitat structure and anthropogenic disturbance differently affected lifetime reproductive success among morphs (Krüger 2002). The morph‐specific differences that we observe in lifetime reproductive success across European populations in this study could thus also be associated with local environmental conditions. In contrast to LRS, in this study, BLS did not vary across morphs within these populations. This is in line with a lack of morph‐specific lifespan differences in buzzards, where the main predictor of lifespan for all individuals was weather conditions (e.g., rainfall and temperature; Krüger 2002). Hence, environmental variation across populations could lead to modifications in life‐history traits that may be morph‐specific in terms of reproductive success. There are several nonexclusive possible explanations ranging from ecological to physiological processes. For example, the morphs may differ in their food preference; pale male tawny owls are more specialised in mammalian prey, while the brown‐reddish ones are more generalist (Karell et al. 2021) which could result in morph‐specific breeding output across the range. Similar dietary differences between morphs have been found in other birds of prey (Roulin 2004b; Avilés et al. 2023) and reptiles (Scali et al. 2015). In this study, males could not be included, but due to the diversification in parental roles in this species and the observed male morph‐specific diet (Karell et al. 2021), including male individuals in future studies could potentially strengthen our understanding of the observed patterns and provide additional insights into the dynamics of nest success. Furthermore, the production of (phaeo)melanin is physiologically linked to a suite of other traits (Ducrest et al. 2008) that have implications for fitness. Previous studies (Karell et al. 2021; Morosinotto et al. 2020; Roulin 2004b; Scali et al. 2015; Avilés et al. 2023; Ducrest et al. 2008; Karell et al. 2011b, 2017; Morosinotto et al. 2022) support the idea that tawny owl morphs might be adapted to varying environmental conditions due to life‐history traits linked to melanin‐based colouration. Colour polymorphism may facilitate ecological diversification across populations, with environmental components shaping morph‐specific fecundity benefits (rather than survival differences). Tawny owl colour morphs differ in their immune defences, highlighting that immune function is associated with melanin production (Karell et al. 2011b). This link suggests that a morph might be able to cope better with infections, allowing it to have better survival and reproductive success under certain circumstances. Additionally, morph‐specific differences have been observed in telomere dynamics and rates of senescence among breeding adults, and these had consequences also for offspring telomere length and overall fitness (Karell et al. 2017; Morosinotto et al. 2022). Fledgling mass is also colour morph‐specific (Morosinotto et al. 2020), with brown individuals able to raise heavier offspring than grey ones, a factor that can affect juvenile survival and future reproductive potential of the chicks.

Our key finding was that reproductive performance varies between the study populations and two of these metrics (lifetime egg production and lifetime reproductive success) vary in a colour morph‐specific manner across populations covering a substantial part of the species' range. LEP and LRS largely correspond and thus differences in lifetime fitness appear to stem mainly from differences in egg production depending on the morph in a population‐specific manner. Identifying such patterns relies on substantial efforts in individual‐based data collection by several researchers. A significant challenge in studying colour polymorphism across different populations is the lack of a common colour‐scoring method (Soares et al. 2001; Robinson et al. 2024). The absence of a universal scale complicates the comparison of colour morph traits between populations. Adopting a standardised system as we did in this study would not only enhance the repeatability and reliability of colour assessments but also facilitate more precise and meaningful comparisons of colour morph variation across different populations. Addressing this gap is crucial for advancing our understanding of the evolutionary and ecological significance of colour. In this study, data collectors used their own colour‐scoring method, which we show to be a repeatable way of colour scoring in most populations. Furthermore, we demonstrate that these population‐specific colour morph scores, when compared to a dichotomous score of an independent observer based on pictures taken, span a similar range (from the lightest colour shade to the darkest one) in all populations. Nevertheless, future work would benefit from applying a single approach to determine tawny owl plumage colouration or developing a protocol for photographing individuals, allowing for consistent colour scoring using a specific method. In particular, there is a relatively large number of individuals here divided into ‘intermediate colour morphs’ (i.e., scores other than the two extremes) ranging from 1 to up to 6 classes, depending on the population. More precise information on where individuals fall within this colour gradient could facilitate cross‐population comparisons.

This work enhances our comprehension of how variation (i.e., different phenotypes) persists for heritable traits under selection. In particular, our findings highlight the complexity of reproductive strategies between different populations and within single populations over large spatial scales, suggesting that factors influencing egg and fledgling production vary across different regions. It also indicates potential adaptations of owls in response to local environmental conditions and resource availability (Emaresi et al. 2014; Morosinotto et al. 2020). Hence, local conditions are an important factor that may affect life‐history and behavioural and ecologically important traits that are linked with resource allocation, dispersal and mating strategies (i.e., fitness) in different phenotypes. By examining how distinct environmental conditions (populations in our case) affect the relationship between heritable polymorphic phenotypes (morphs in our case) and lifetime fitness estimates, our study contributes to a better understanding of how polymorphism can be maintained.

## Author Contributions

**Gian Luigi Bucciolini:** data curation (lead), formal analysis (lead), funding acquisition (equal), investigation (lead), methodology (equal), visualization (lead), writing – original draft (lead), writing – review and editing (lead). **Chiara Morosinotto:** conceptualization (equal), formal analysis (equal), investigation (equal), methodology (equal), supervision (equal), validation (equal), writing – original draft (equal), writing – review and editing (equal). **Jon Brommer:** formal analysis (equal), funding acquisition (equal), investigation (equal), methodology (equal), supervision (equal), validation (equal), writing – original draft (equal), writing – review and editing (equal). **Al Vrezec:** data curation (equal), funding acquisition (equal), validation (supporting), writing – review and editing (equal). **Peter Ericsson:** data curation (equal), funding acquisition (equal), validation (supporting), writing – review and editing (equal). **Lars‐Ove Nilsson:** data curation (equal). **Karel Poprach:** data curation (equal), funding acquisition (equal), validation (supporting), writing – review and editing (equal). **Ingar Jostein Øien:** data curation (equal), funding acquisition (equal), validation (supporting), writing – review and editing (equal). **Patrik Karell:** conceptualization (lead), formal analysis (equal), funding acquisition (equal), investigation (equal), methodology (equal), supervision (equal), validation (equal), writing – original draft (equal), writing – review and editing (equal).

## Consent

All authors have reviewed and approved the manuscript and consent to its submission.

## Conflicts of Interest

The authors declare no conflicts of interest.

## Data Availability

The datasets generated and/or analysed during the current study are available in the Mendeley data repository, web link for datasets https://data.mendeley.com/datasets/bjh96g9j6m/1 (doi: 10.17632/bjh96g9j6m.1).
